# Identification of a prognostic and therapeutic immune signature associated with hepatocellular carcinoma

**DOI:** 10.1186/s12935-021-01792-4

**Published:** 2021-02-10

**Authors:** Yanan Peng, Chang Liu, Mengting Li, Wenjie Li, Mengna Zhang, Xiang Jiang, Ying Chang, Lan Liu, Fan Wang, Qiu Zhao

**Affiliations:** 1 Department of Gastroenterology, Zhongnan Hospital of Wuhan University, Wuhan, China; 2 Hubei Clinical Center and Key Lab of Intestinal and Colorectal Diseases, Wuhan, China; 3 Department of Obstetrics, Renmin Hospital of Wuhan University, Wuhan, China

**Keywords:** Immune risk signature, Hepatocellular carcinoma, Immune cells infiltration, Tumor immune microenvironment, Differentially expressed immune-related genes, Immune checkpoint inhibitor

## Abstract

**Background:**

Hepatocellular carcinoma (HCC) is one of the most prevalent and inflammation-associated cancers. ﻿The tumor microenvironment (TME) plays an essential role in HCC development and metastasis, leading to poor prognosis. The overall TME immune cells infiltration characterizations mediated by immune-related genes (IRGs) remain unclear. In this study, we aimed to investigate whether immune-related genes could be indicators for the prognosis of HCC patients and TME cell infiltration characterization as well as responses to immunotherapy.

**Methods:**

We obtained differentially expressed immune-related genes (DE IRGs) between normal liver tissues and liver cancer tissues from The Cancer Genome Atlas (TCGA) database. To identify the prognostic genes and establish an immune risk signature, we performed univariable Cox regression survival analysis and the Least Absolute Shrinkage and Selector Operation (LASSO) regression based on the DE IRGs by robust rank aggregation method. Cox regression analysis was used to identify independent prognostic factors in HCC. We estimated the immune cell infiltration in TME via CIBERSORT and immunotherapy response through TIDE algorithm.

**Results:**

We constructed an immune signature and validated its predictive capability. The immune signature included 7 differentially expressed IRGs: BIRC5, CACYBP, NR0B1, RAET1E, S100A8, SPINK5, and SPP1. The univariate and multivariate cox analysis showed that the 7-IRGs signature was a robust independent prognostic factor in the overall survival of HCC patients. The 7-IRG signature was associated with some clinical features, including gender, vascular invasion, histological grade, clinical stage, T stage. We also found that the 7-IRG signature could reflect the infiltration characterization of different immunocytes in the tumor microenvironment (TME) and had a good correlation with immune checkpoint molecules, revealing that the poor prognosis might be partly due to immunosuppressive TME. The Tumour Immune Dysfunction and Exclusion (TIDE) analysis data showed that the 7-IRG signature had great potential for indicating the immunotherapy response in HCC patients. The mutation analysis demonstrated a significant difference in the tumor mutation burden (TMB) between the high- and low-risk groups, partially explaining this signature's predictive value.

**Conclusion:**

In a word, we constructed and validated a novel, immune-related prognostic signature for HCC patients. This signature could effectively indicate HCC patients' survival and immunotherapy response. And it might act as potential immunotherapeutic targets for HCC patients.

## Background

Hepatocellular carcinoma (HCC), with 42,810 cases diagnosed and 30,160 deaths in the United States in 2020 [1], is the sixth most widespread neoplasm and the third leading cause of cancer death [2]. The incidence of HCC is gradually increasing, and liver cirrhosis is the leading cause of death. Treatment of HCC requires multidisciplinary experts, including hepatologists, surgeons, radiologists, pathologists, and oncologists [3, 4]. However, it is still challenging to prevent advanced HCC, and systemic chemotherapy has toxic side effects and no survival benefits [5, 6]. Recently, immunotherapy is a very promising therapy in many advanced cancers, particularly in those induced by viruses [7–9]. Most HCC patients in China are infected with the hepatitis B virus, indicating that HCC patients perhaps be suitable for immunotherapy.

The tumor microenvironment (TME) consists of the stromal and immune cells which interact with or infiltrate a given tumor [10]. In HCC, TME is immunosuppressive and ﻿promotes immune tolerance and evasion by various mechanisms, promoting tumor proliferation, invasion, and metastasis [10]. Recently, ﻿many studies have demonstrated that the tumor immune microenvironment (TIME) containing the effector of CD8 + , CD4 + cells, regulatory T cells, and dendritic cells (DCs) could affect the efficacy of immune checkpoint inhibitors(ICIs) [11, 12]. Thus, to figure out the cause that influences the immunosuppression of TME and the clinical response of ICIs, we need to explore some immunological genes affecting the abundance of immune cells in TME. Targeted research may significantly change the clinical outcome of HCC.

Some immune checkpoint molecules are often the targets of immunotherapy. These immune checkpoint molecules include programmed cell death protein 1 (PD-1), cytotoxic T lymphocyte-associated antigen 4 (CTLA-4), PD-1 ligand 1 (PD-L1), B7 homolog 3 (B7-H3), and others. The dysregulation of immune checkpoint molecules could inhibit anti-tumor immune responses in many cancers, including liver cancer, resulting in cancer development and progression [13]. Ipilimumab (the CTLA4 inhibitor) and nivolumab/pembrolizumab (the PD-1 inhibitor) have demonstrated great survival benefits for HCC [14, 15]. Nevertheless, most of the immune checkpoint inhibitors (ICIs) are effective in only a portion of patients, according to a phase I/II study. For instance, Nivolumab and Pembrolizumab (PD-1 inhibitor) have approximately 16–20% of the response rate in advanced HCC patients [16, 17]. Therefore, it is necessary to investigate predictive biomarkers to indicate checkpoint inhibitor-based immunotherapy responsiveness. Several studies reported that immune-related genes (IRGs) or TME could serve as promising biomarkers for evaluating survival in multiple cancers [18–20]. Thus, it is indispensable to establish a robust gene signature of HCC based on IRGs or TME to indicate the prognosis and the immunotherapy response of HCC patients [21].

This study aimed to build a novel immune-related risk signature with powerful predictive capability based on IRGs or TIME to enhance HCC prognostic prediction by comprehensive genomic data analysis. We carried out a univariate Cox proportional hazard regression analysis to explore the relationship between overall survival (OS). We screened  differentially expressed IRGs (DE IRGs) in the training set. These DE IRGs were then subjected to the least absolute shrinkage and selection operator (LASSO) Cox regression and multivariate Cox proportional hazard regression analysis to acquire 7 IRGs to establish the risk signature. We calculated the individualized risk score with coefficients, dividing patients into a high-risk or low-risk group according to the risk score's median cutoff. Survival analysis and ROC curve estimate the risk signature's predictive value in the internal and external dataset. Univariate and multivariate cox analysis proved that the risk signature was an independent prognostic factor. The risk signature has a positive correlation with some clinical features. We utilized CIBERSORT and ESTIMATE algorithms to assess the infiltration levels of immune cells in TME and the activity of immune cells and stromal cells, respectively.

Moreover, we found that the immune checkpoint molecules were differentially expressed in risk groups and had a good correlation with the 7 IRGs. The Tumour Immune Dysfunction and Exclusion (TIDE) algorithm estimates the immune-checkpoint inhibitors (ICIs) response of TCGA HCC patients. The tumor mutation burden (TMB) is associated with identifying neoantigens arising in a tumor and represents a predictive indicator of responsiveness to Immunotherapy [22]. Thus, we also performed the TMB analysis difference between the high- and low-risk group.

In conclusion, We established a novel 7-IRG risk signature correlated to the survival of HCC patients. The signature might indicate the prognosis and the ICIs response of HCC patients. It may be beneficial to provide new ideas for in-depth immunological studies and more specific immunotherapy for HCC patients.

## Methods

### Patient data acquisition

Samples in datasets that meet the following inclusion criteria were included in this study: (1) the sample with both mRNA sequencing data and clinical information; (2) the sample with prognosis information. HCC patients with the transcriptomic RNA-sequencing data were downloaded from The Cancer Genome Atlas (TCGA) data portal (https://portal.gdc.cancer.gov/). Then the resting 372 TCGA-HCC samples were used. Meanwhile, GSE14520 [23] (n = 242) downloaded from GEO (https://www.ncbi.nlm.nih.gov/geo/) database and LIRI-JP (n = 232) retrieved from International Cancer Genome Consortium (ICGC) database (https://icgc.org/) were chosen for external validation.

A comprehensive list of IRGs was acquired from the Immunology Database and Analysis Portal (ImmPort) database (https://immport.niaid.nih.gov), which accurately and timely updates the immunology data and shares the data for immunologic research [24]. The database provides a list of IRGs for cancer researchers, and these genes actively participate in the process of immune activity.

### Identification of differentially expressed genes (DEGs)

We screened differentially expressed genes (DEGs) between HCC and normal tissues using the edgeR package in the R software. An adjusted *p*-value < 0.05 and |log2 (FC)| value > 1 was considered significant. To further screen the differentially expressed immune-related genes (DE IRGs) participating in the development and progression of HCC, we took the intersection between the DEGs and the IRGs (mentioned above).

### Gene set enrichment analysis

We conducted functional enrichment analyses to explore the possible molecular mechanisms of DE IRGs. We used the DAVID database to perform gene ontology (GO) and Kyoto Encyclopedia of Genes and Genomes (KEGG) analysis of DE IRGs, and false discovery rate (FDR) < 0.05 were considered significantly enriched. We used the R package "ggplot2" to realize the visualization of enrichment analysis.

### Development and verification of the immune-related signature for HCC

The 372 HCC samples were randomly divided into the training group (n = 186) and the testing group (n = 186). We identified the immune-related risk signature and established a prognostic immune-related risk model in the training set. To screen survival-related DE IRGs for HCC patients, we performed a univariate Cox proportional hazard regression analysis to explore the relationship between OS and DE IRGs in the training set. Survival-related IRGs were verified with *p* < 0.05. We conducted a least absolute shrinkage and selection operator (LASSO) regression analysis to evaluate the identified survival-associated IRGs combined with the expression profiles in training set to minimize overfitting and find the best gene model using the R package "glmnet". We calculated the individualized risk score with coefficients, divided patients into a high-risk or low-risk group according to the risk score's median cutoff. The risk score was established with the following formula: Risk score = expression of Gene 1*coefficient + expression of Gene 2 *coefficient + …expression of Gene n * coefficient [25]. We constructed the immune-related risk signature model to predict the prognosis of HCC patients. We used the R package "survival" and "survminer" to investigate the optimal cutoff of risk score and draw the Kaplan–Meier survival curve. Kaplan–Meier survival curves showed the difference in OS between the high-risk and low-risk groups, which were stratified based on the immune signature. We calculated the area under the curve (AUC) with R package "survivalROC" [26] to evaluate the time-dependent prognostic value of the gene signature [27]. A two-sided log-rank P < 0.05 was considered significant for survival analysis. We validated the predictive capability of the established risk signature in the testing set and the total set. The concordance index (C-index) was calculated to investigate the prediction accuracy of the immune risk signature. Then, we also validated the risk signature in GEO and ICGC dataset, respectively.

Clinical features including age, gender, BMI, AFP, vascular invasion, clinical stage, histological grade, and tumor-node-metastasis (TNM) status were collected from the TCGA database. After that, we performed both univariate and multivariate Cox regression analyses to verify whether the signature predicted prognosis independently from these clinical features. A value of *p* < 0.05 was considered significant statistically.

### Estimate the difference of tumor-infiltrating immune cells and immune checkpoint inhibitors response between high- and low-risk Groups

We used the CIBERSORT algorithm to assess the proportions of 22 types of infiltrating immune cells based on TCGA gene expression RNA-sequencing data following the previously reported procedure [28]. CIBERSORT is an influential deconvolution algorithm, utilizing gene expression data with a predefined immune signature matrix to estimate the fraction of 22 human tumor-infiltrating immune cells within a given sample [28]. The sum of all estimate immune cell type fractions equals 1 for each HCC sample [29].

We applied the "Estimation of Stromal and Immune cells in Malignant Tumours using Expression data" (ESTIMATE) algorithm to assess the immune scores, stromal scores, estimate scores, and tumor purity for each HCC sample [30]. Furthermore, Tumour Immune Dysfunction and Exclusion (TIDE) algorithm was used to estimate the immune-checkpoint inhibitors (ICIs) response of HCC patients [31].

### Mutation analysis

We downloaded the available mutation data of TCGA HCC patients from the TCGA data portal(https://portal.gdc.cancer.gov/). The somatic variant data was stored as Mutation Annotation Format (MAF), and we used the maftools [32] to analyze the mutation data of HCC samples. We calculated the tumor mutation burden (TMB) score for every HCC patient. The TMB score was calculated as follows: (total mutation/total covered bases) × 10^6 [33].

### Cell lines and cell culture

Human normal liver cell line LO2 (CRL-12461) and human liver cancer cell lines HepG2 (HB-8065), Hep3B (HB-8064) were purchased from the American Type Culture Collection (ATCC; Manassas, VA, USA) in June 2019. Another liver cancer cell line SMMC-7721 was purchased from the Institute of Cell Research, Shanghai Academy of Health Sciences, China. Cells were cultured at 37 °C with 5% CO2 in Dulbecco’s Modifed Eagle’s Medium (DMEM) containing 10% Fetal bovine serum (FBS) and 1% penicillin/streptomycin (Gibco, USA).

### Real-time quantitative PCR

The primers are as follows:

BIRC5 gene 5′-GACCACCGCATCTCTACATTCA-3′ (sense) and 5′-CTCGTTCTCAGTGGGGCAGT—3′ (anti-sense).

CACYBP gene 5′-CTGACCCAGGTTGAAAAGGAGT-3′ (sense) and 5′-GCTTCTCTCTTGATTCCACCCA -3′ (anti-sense).

NR0B1 gene 5′-GGGGACCGTGCTCTTTAACC-3′ (sense) and 5′-TCGATGAATCTGTCATGGGGC-3′ (anti-sense).

S100A8 gene 5′-TGCTAGAGACCGAGTGTCCT-3′ (sense) and 5′-GCCACGCCCATCTTTATCAC-3′ (anti-sense).

RAET1E gene 5′-AGCTTCCTGCCTGTTACTCT-3′ (sense) and 5′-GGTCAATTCTCCCCAAGTGC-3′ (anti-sense);

SPINK5 gene 5′-ACCCTGTTCGAGGCCCAT-3′ (sense) and 5′-ATTCCCAAAGCTGGAGAAGAATG-3′ (anti-sense);

SPP1 gene 5′-AGGCTGATTCTGGAAGTTCTGAG-3′ (sense) and 5′-GGCAGGTCCGTGGGAAAATC-3′ (anti-sense).

All reactions were performed on the Roche LightCycler® 96 Instrument using following cycling parameters, 95 °C for 2 min, followed by 40 cycles of 95 °C for 15 s, 60 °C for 45 s.

### Statistical analysis

Continuous variables were summarized as mean ± SE or median; categorized variables were described by frequency (n) and proportion (%). Differences among variables were tested using t-tests, nonparametric tests, chi-square tests. We performed univariate and multivariate cox regression analysis to estimate the predictive power of the immune-related risk signature and clinical features. We used Graphpad prism 9.0, SPSS, and R software, version 4.0.0, to conduct statistical analyses. The volcano plot and the heatmap were generated using the R package "ggplot2" and "pheatmap", respectively.

## Results

### HCC patients' data preparation

Hepatocellular carcinoma RNA-sequencing expression profiles and clinical information for 372 patients were publicly available and downloaded from the TCGA database. We divided patients randomly into the training set (n = 186) and the testing set (n = 186). There were no significant differences (*p* > 0.05) in clinical variables between the training and testing sets. Table 1 shows the overall study design.

**Table 1:** Clinical variables in the training and testing sets

Variables	Total set (n = 372)	Training set (n = 186)	Testing set (n = 186)	p value	Methods
Survival time (days)	555.0 (232.25–1085.0)	555.5 (324.0–1088.25)	555.0 (320.0–1085.75)	0.94	Mann–Whitney U test
Vital status					
Live	246 (66.13%)	117 (62.90%)	129 (69.35%)	0.189	χ2 test
Dead	126 (33.87%)	69 (37.10%)	57 (30.65%)		
Age (years)					
≤ 65	234 (62.9%)	120 (64.52%)	114 (61.29%)	0.52	χ2 test
> 65	138 (37.1%)	66 (35.48%)	72 (38.71%)		
Gender					
Male	251 (37.1%)	124 (66.67%)	127 (68.28%)	0.74	χ2 test
Female	121 (32.53%)	62 (33.33%)	59 (31.73%)		
BMI (kg/cm^2^)					
< 25	178 (47.85%)	85 (45.70%)	93 (50.00%)	0.35	χ2 test
≥ 25	157 (42.20%)	85 (45.70%)	72 (38.71%)		
NA	37 (9.95%)	16 (8.6%)	21 (11.29%)		
Histological grade					
G1	55 (14.78%)	29 (15.59%)	26 (13.98%)	0.925	χ2 test
G2	178 (47.85%)	86 (46.24%)	92 (49.46%)		
G3	122 (32.8%)	61 (32.8%)	61 (32.80%)		
G4	12 (3.23%)	7 (3.76%)	5 (2.69%)		
NA	5 (1.34%)	3 (1.61%)	2 (1.08%)		
Clinical stage					
I	172 (46.24%)	86 (46.24%)	86 (46.24%)	0.788	χ2 test
II	86 (23.12%)	39 (20.97%)	47 (25.27%)		
III	85 (22.85%)	44 (23.66%)	41 (22.04%)		
IV	5 (1.34%)	3 (1.61%)	2 (1.08%)		
NA	24 (6.45%)	14 (1.61%)	10 (5.38%)		
T stage					
T1	182 (48.92%)	90 (48.39%)	92 (49.46%)	0.806	χ2 test
T2	94 (25.27%)	44 (23.66%)	50 (26.88%)		
T3	80 (21.51%)	44 (23.66%)	36 (19.35%)		
T4	13 (3.49%)	7 (3.76%)	6 (3.23%)		
Tx	3 (0.81%)	1 (0.54%)	2 (1.08%)		
M stage					
M0	267 (71.77%)	132 (70.97%)	135 (72.58%)	0.593	χ2 test
M1	4 (1.08%)	3 (1.61%)	1 (0.54%)		
Mx	101 (27.15%)	51 (27.42%)	50 (26.88%)		
N stage					
N0	253 (68.01%)	124 (66.67%)	129 (69.35%)	0.467	χ2 test
N1	4 (1.08%)	1 (0.54%)	3 (1.61%)		
Nx	115 (30.91%)	61 (32.8%)	54 (29.03%)		
Adjacent hepatic tissue inflammation extent					
None	118 (31.72%)	64 (34.41%)	54 (29.03%)	0.12	χ2 test
Mild	100 (26.88%)	48 (25.81%)	52 (27.96%)		
Severe	17 (4.57%)	4 (2.15%)	13 (6.99%)		
NA	137 (36.83%)	70 (37.63%)	67 (36.02%)		
Child–pugh classification grade					
A	217 (58.33)	103 (3.76%)	114 (61.29%)	0.09	χ2 test
B	21 (5.65%)	7 (3.76%)	14 (7.53%)		
C	1 (0.27%)	0 (0.00%)	1 (0.54%)		
NA	133 (35.75%)	76 (40.86%)	57 (30.65%)		
AFP (ng/ml)					
< 300	213 (57.26%)	103 (55.38%)	110 (59.14%)	0.76	χ2 test
≥ 300	65 (17.47)	34 (18.28%)	31 (16.67%)		
NA	94 (25.27%)	49 (26.34%)	45 (24.19%)		
Fibrosis ishak score					
0-No fibrosis	75 (20.16%)	44 (23.66)	31 (16.67%)	0.38	χ2 test
1,2-Portal fibrosis	31 (8.33%)	16 (8.6%)	15 (8.06%)		
3,4-Fibrous speta	28 (7.53%)	15 (8.06)	13 (6.99%)		
5-Nodular formation and incomplete cirrhosis	9 (2.42%)	4 (2.15%)	5 (2.69%)		
6-established cirrhosis	70 (18.82%)	28 (15.05%)	42 (22.58%)		
NA	159 (42.74%)	79 (42.47%)	80 (43.01%)		
Hepatic carcinoma risk factor					
No history of primary risk factors	86 (23.12%)	46 (24.73%)	40 (21.51%)	0.14	χ2 test
Alcohol consumption	113 (30.38%)	57 (30.65%)	56 (30.11%)		
Hepatitis B	100 (26.88%)	42 (22.58%)	58 (31.18%)		
Hepatitis C	54 (14.52%)	24 (12.90%)	30 (16.13%)		
Non-Alcoholic fatty liver disease	20 (5.38%)	16 (8.60%)	4 (2.15%)		
Hemochromatosis	6 (1.61%)	3 (1.61%)	3 (1.61%)		
Others	20 (5.38%)	10 (5.38%)	10 (5.38%)		
NA	18 (91.13%)	8 (4.3%)	10 (5.38%)		
Vascular invasion					
None	207 (55.65%)	98 (52.69%)	109 (58.06%)	0.33	χ2 test
Mico	93 (25.00%)	45 (24.19%)	48 (25.81%)		
Macro	16 (4.30%)	10 (5.38%)	6 (3.23%)		
NA	56 (15.05%)	33 (17.74%)	23 (12.37%)		
Histological diagnosis					
Hepatocellular carcinoma	358 (96.24%)	178 (95.70%)	180 (96.77%)	0.69	χ2 test
Cholangiocarcinoma	7 (1.88%)	4 (2.15%)	3 (1.61%)		
Clear cell carcinoma	4 (1.08%)	3 (1.61%)	1 (0.54%)		
Fibrolamellar carcinoma	3 (0.81%)	1 (0.54%)	2 (1.08%)		

### Identification of HCC differentially expressed immune-related genes

**Fig. 1:**
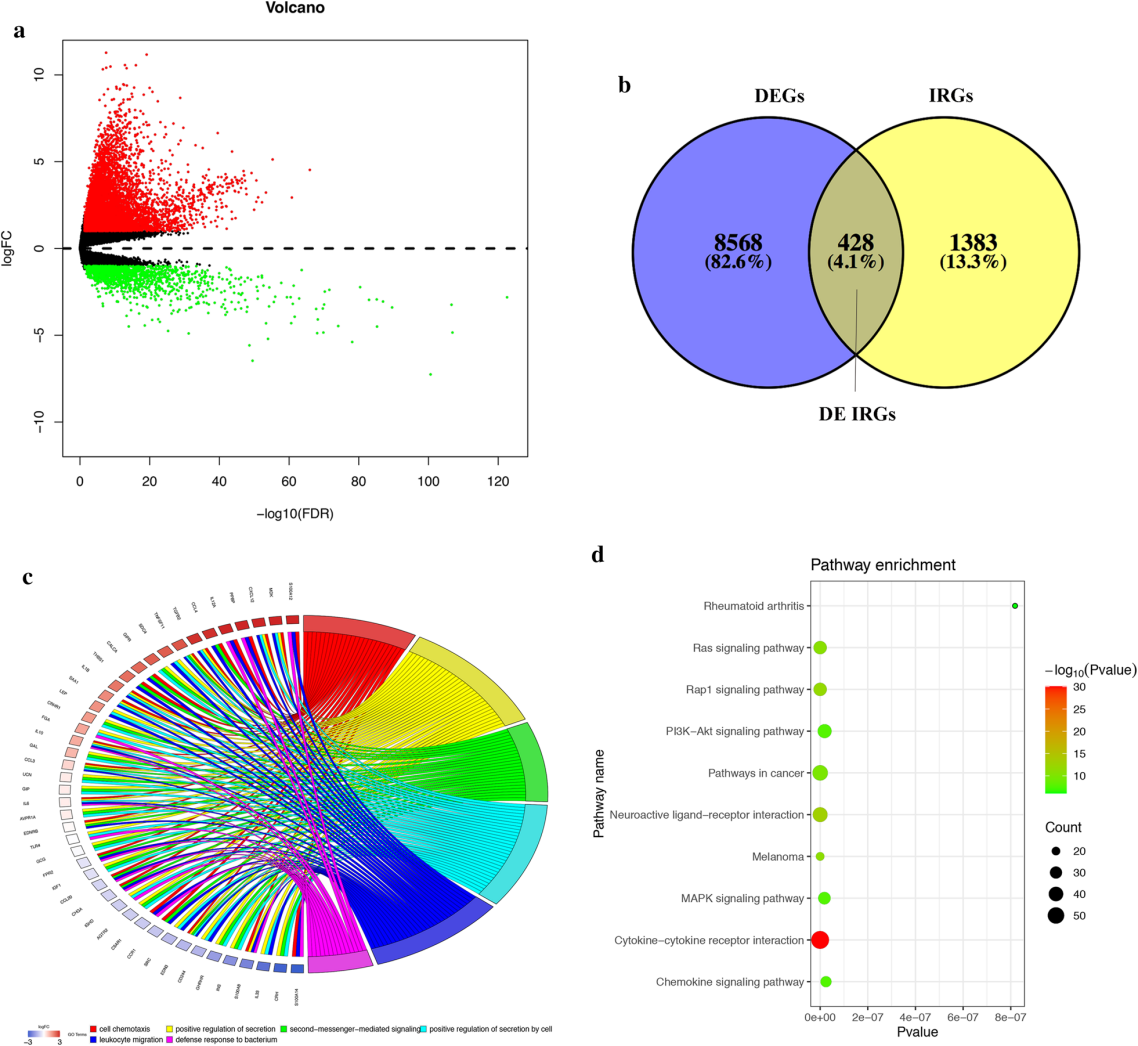
Identification of DE IRGs and functional enrichment analysis of DE IRGs. **a** Volcano plot of DEGs between HCC and non-tumor liver tissues based on the TCGA database. **b** Venn diagram for the intersections between HCC DEGs and IRGs. **c** GO analysis of DE IRGs. **d** The top 10 most enriched KEGG pathways

Based on the adjusted P-value < 0.05 and | log2 (fold change) |> 1, we obtained 8996 differentially expressed genes (DEGs) in the TCGA dataset: 7444 genes were up-regulated and 1552 downregulated. The intersection of the immune-related genes and DEGs in HCC identified 428 differentially expressed immune-related genes (DE IRGs) (Fig. 1a, b). We extracted DE IRGs to perform enrichment analyses by using the DAVID database. Figure 1c depicted the six most highly enriched terms for gene ontology (GO) analysis. The most enriched GO terms were "cell chemotaxis", "positive regulation of secretion", "second-messenger-mediated signaling", "positive regulation of secretion by cell", "leukocyte migration", and "defense response to bacterium". The top 10 pathways obtained by KEGG analysis were: "cytokine-cytokine receptor interaction", "neuroactive ligand-receptor interaction", "Rap1 signaling pathway", "melanoma", "Ras signaling pathway", "Pathways in cancer", "MAPK signaling pathway", "PI3K-Akt signaling pathway", "Chemokine signaling pathway" and "Rheumatoid arthritis" (Fig. 1d). PI3K − Akt and MAPK signaling pathways are pivotal in regulating immune responses [34].

### Development of immune-related risk signature

**Fig. 2:**
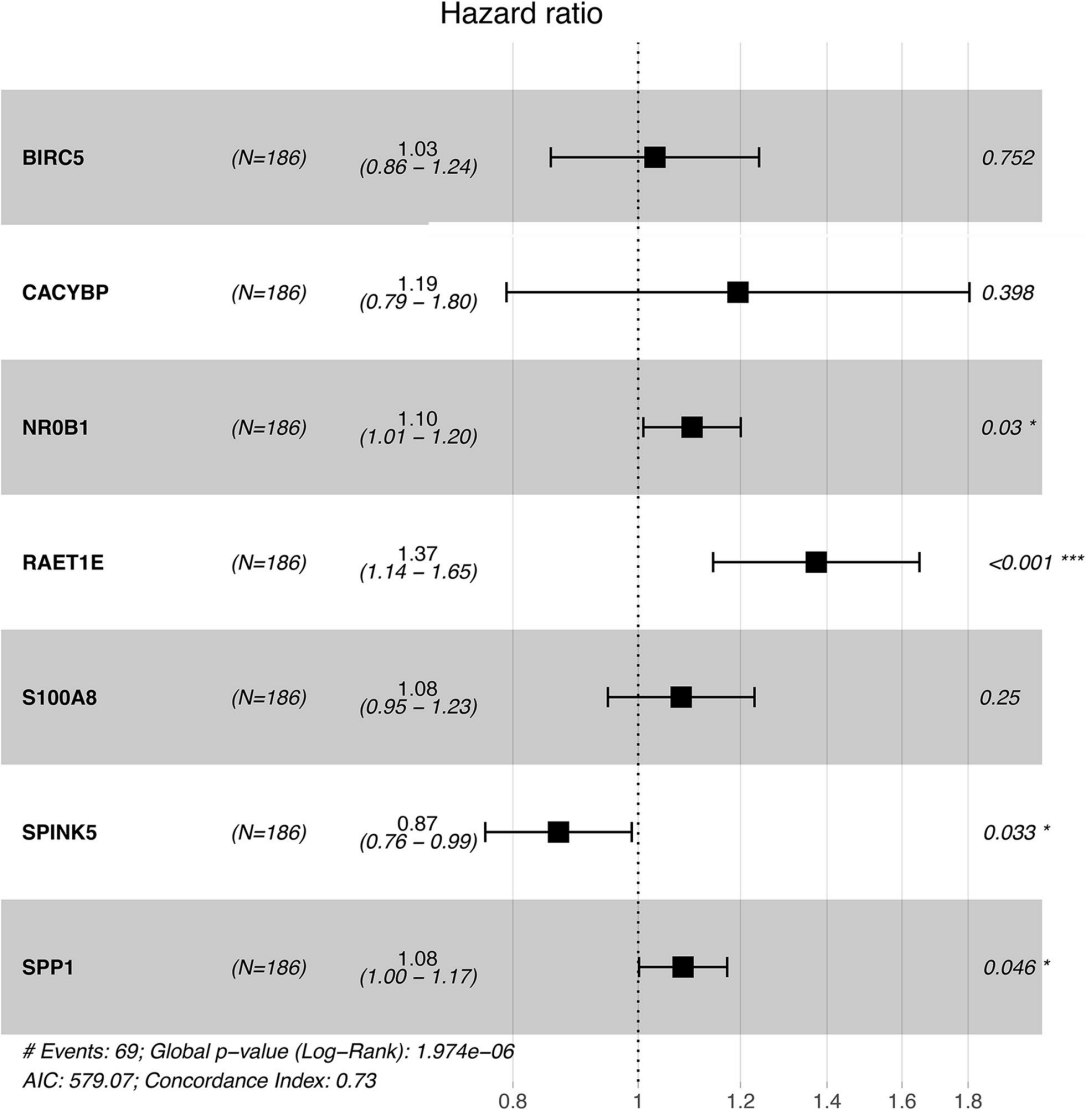
Forest plot demonstrating the multivariable Cox model results of 7 immune-related signature genes

To investigate the prognostic value of these 428 DE IRGs, we performed a univariate Cox regression analysis. And we identified 51 DE IRGs, which were significantly related to the overall survival of HCC patients in the training set (*p* < 0.05). We conducted a LASSO Cox regression in the training set with expression profiles of the overall survival associated with DE IRGs and identified 7 prognostic IRGs (Fig. 2). To establish a clinically applicable risk assessment model, we built the immune signature based on the expression of these 7 DE IRGs and their corresponding coefficient obtained from multivariate Cox regression (Table 2). The formula is risk score = (0.07661*S100A8) + (0.02989*BIRC5) + (0.17778*CACYBP) + (0.09598*NR0B1) + (0.31729*RAET1E) + (-0.141195*SPINK5) + (0.07996*SPP1). Six IRGs were associated with high risk (S100A8, BIRC5, CACYBP, NR0B1, RAET1E, SPP1, Coefficient > 0), and SPINK5 was protective with Coefficient < 0 (Table 2). Additional file 1: Table S1 lists the detailed information of these 7 DE IRGs.

**Table 2:** Coefficients and multivariable Cox model results of 7 immune-related signature genes

Gene	Regulation	LogFC	Coefficient	HR	Z score	p value
S100A8	Down	− 1.0998	0.07661	1.08	1.150	0.250267
BIRC5	Up	4.1028	0.02989	1.03	0.316	0.752159
CACYBP	Up	1.0713	0.17778	1.19	0.845	0.398157
NR0B1	Up	6.4852	0.09598	1.10	2.167	0.030230
RAET1E	Up	1.8010	0.31729	1.37	3.383	0.000717
SPINK5	Up	3.2213	− 0.14195	0.87	− 2.133	0.032963
SPP1	Up	4.3101	0.07996	1.08	1.996	0.045903

**Fig. 3:**
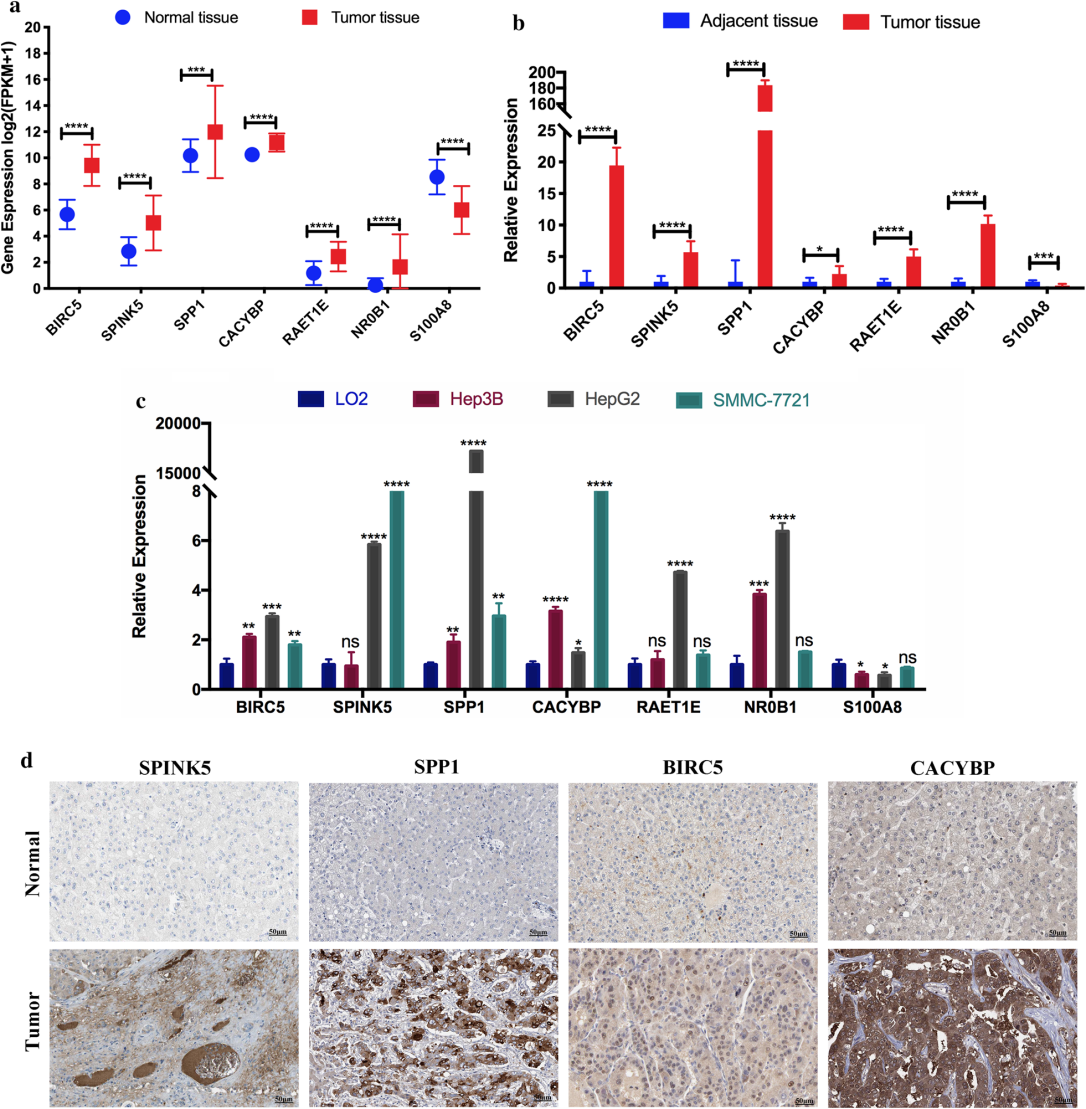
Expression analysis of 7 IRGs. **a** The expression of 7 IRGs in the normal and tumor tissues based on the TCGA-HCC dataset. **b** Validation of the expression of 7 IRGs in 7 pairs of liver tumor tissues and corresponding adjacent tissues. **c** Validation of the expression of 7 IRGs in the liver normal cell line, LO2, and 3 liver cancer cell lines, Hep3B, HepG2, and SMMC-7721. **d** The immunohistochemical staining results showed significant differences of 4 IRGs at the protein expression between liver normal and tumor tissues. *P < 0.05; **P < 0.01; ***P < 0.001; ns, not significant

As shown in Fig. 3a, BIRC5, SPINK5, SPP1, CACYBP, RAET1E, and NR0B1 were significantly up-regulated, and S100A8 was significantly down-regulated in hepatocellular carcinoma samples based on TCGA-HCC dataset. To validated the expression stability of these 7 DE IRGs, we examined their expression in 7 pairs of liver tumor tissues and corresponding adjacent tissues (Fig. 3b). We also compared their expression in one human normal liver cell line, LO2, with three human liver cancer cell lines, HepG2, Hep3B, SMMC-7721 (Fig. 3c). We found that the expression difference of these 7 IRGs in HCC tissue and liver cancer cell lines was consistent with those from the TCGA dataset. After carefully screening the (Human Protein Atlas) HPA database, we only acquired the immunohistochemical results of CACYBP, BIRC5, SPP1, and SPINK5 in the HPAdatabase. We could qualitatively observe the noticeable expression difference of these 4 DE IRGs between normal and HCC samples at the protein levels (Fig. 3d).

**Fig. 4:**
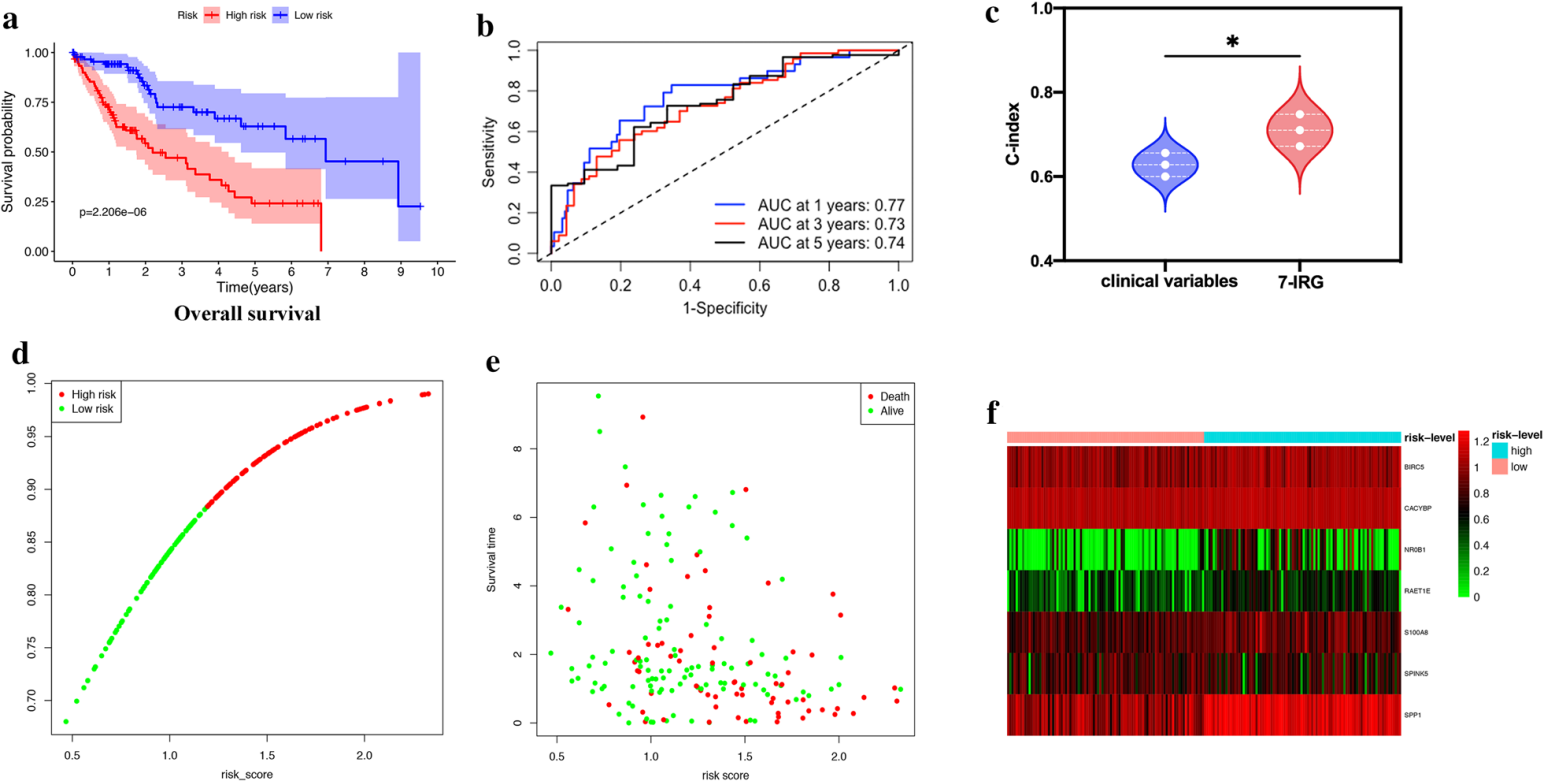
Development of the prognostic signature based on 7 IRGs in the training set. **a** Overall survival (OS) of HCC patients in high- and low-risk groups. **b** Time- ROC curve of the 7-IRG prognostic signature. **c** The concordance index (C-index) of the clinical variates ﻿versus 7-IRG signature to evaluate prognostic accuracy for OS prediction. **d** Risk score distribution, **e** survival status, and **f** heatmap of expression profiles in high- and low-risk groups. *p < 0.05; ** *p* < 0.01; *** *p* < 0.001; ns, not significant

Then, we computed the risk score for each patient in the training group and set the median of the risk score to classify patients into a high-risk group (n = 92) or a low-risk group (n = 92). The low-risk group had significantly better overall survival (OS) than the high-risk group (Fig. 4a, p = 2.206e–06, log-rank test). ROC curve analysis of OS based on the 7-IRG risk signature indicated acceptable discrimination with AUC of 0.77, 0.73, and 0.74 in predicting 1-, 3-, 5-year overall survival, respectively (Fig. 4b). Then we compared the concordance index (C-index) of clinical variables (including age, gender, and clinical stage) and 7-IRG signature. The prediction accuracy of the 7-IRG signature was better than clinical variables (﻿Fig. 4c, C-index: 0.71 vs. 0.628, *p* = 0.04). We ranked the risk score of patients in the training set and analyzed their distribution in Fig. 4d. Figure 4e depicted the survival status of HCC patients in the training set. The heatmap demonstrated the expression level of these 7 IRGs between low- and high-risk cancer groups (Fig. 4f).

### Internal validation of the predictive capability of the 7-IRG risk signature

**Fig. 5:**
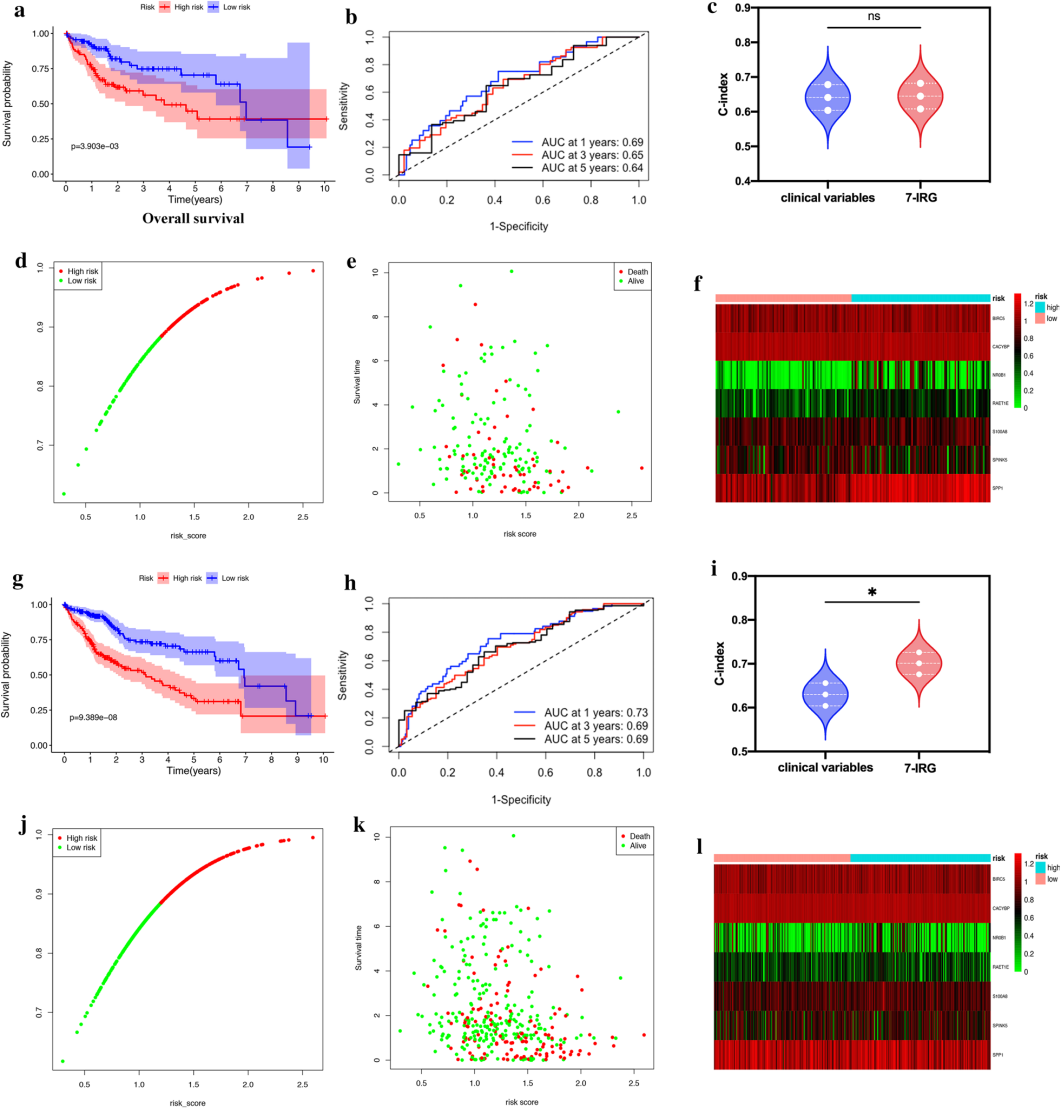
Validation of the 7-IRG prognostic signature in the test and total set. OS of HCC patients in the test (**a**) and total set (**g**). ROC curve of OS according to risk groups in the test (**b**) and total set (**h**). The C-index of clinical variates ﻿and 7-IRG signature in the test (**c**) and total set (**i**). Risk score distribution in the test (**d**) and total set (**j**). Survival status of HCC patients in the test (**e**) and total set (**k**), and heatmap of expression profiles of 7 IRGs between high- and low-risk groups in the test (**f**) and total set (**l**). *p < 0.05; ** *p* < 0.01; *** *p* < 0.001; ns, not significant

To assess the predictive ability of the 7-IRG signature, we verified it and calculated the risk-score for each patient in the testing and total set. We divided all of the patients in the testing set into a high-risk group (n = 93) or a low-risk group (n = 91). Likewise, survival analysis demonstrated that the high-risk group's OS was more unfortunate than the low-risk group (Fig. 5a, *p* = 3.903e–03). In the testing set, ROC curve analysis based on the 7-IRG risk signature indicated the one, three, and five years AUC were 0.69, 0.65, and 0.64, respectively (Fig. 5b). The c-index of the 7-IRG is similar to clinical variables (Fig. 5c, 0.641 vs. 0.645, *p* = 0.901). Figure 5d–f showed the distribution of risk score, survival status, and the expression profile of 7 IRGs in different risk groups. Similarly, patients in the total set were divided into the low-risk (n = 184) or the high-risk (n = 189) group based on 7-IRGs (Fig. 5g–l). Low-risk patients had more prolonged OS than high-risk patients (Fig. 5g, *p* = 9.389e–08). In the total set, the corresponding AUC of 1-, 3-, and 5-year was 0.73, 0.69, and 0.69 (Fig. 5h). The C-index was also better in the 7-IRG than clinical variables (Fig. 5i, 0.701 vs. 0.630, *p* = 0.027). Figure 5j–l depicted the distribution of risk score, survival status, and expression level of 7 IRGs in the total set.

**Fig. 6:**
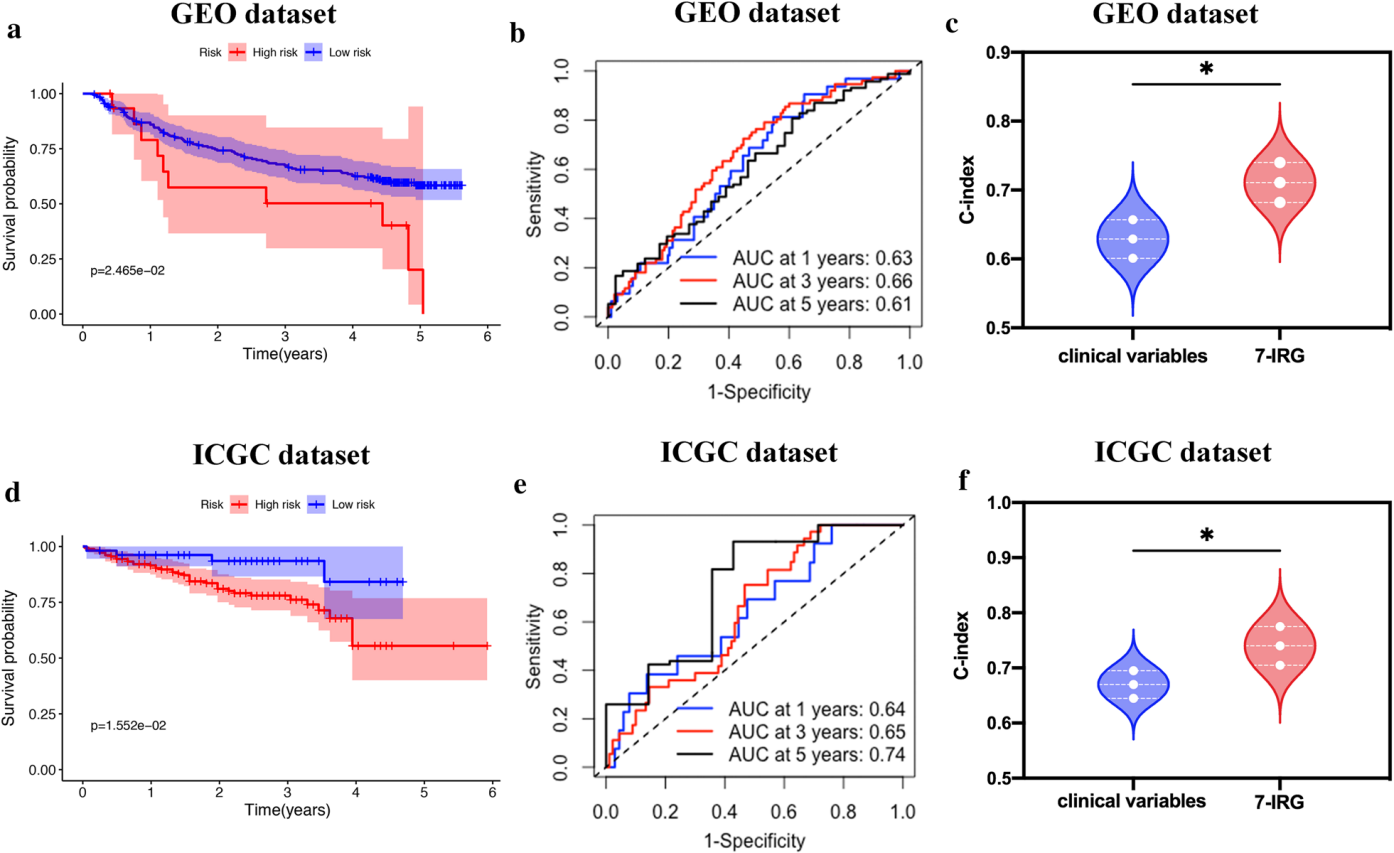
External validation of the prognostic performance of the 7-IRG signature in the GEO and ICGC dataset. **a** OS of HCC patients, **b** time-dependent ROC curve 1-, 3-, and 5-year OS predictions between risk groups, and **c** the C-index of clinical variates ﻿versus 7-IRG signature in GSE14520. **d** Kaplan–Meier curves and **e** ROC curve of OS in ICGC HCC dataset; **f** the C-index for clinical variates and 7-IRG in ICGC HCC dataset

### External validation of the 7-IRG risk signature in GEO and ICGC dataset

To further verify our analysis's stability, we validated the 7-IRG risk signature in the GSE14520 and ICGC (﻿LIRI-JP) dataset, including 242 and 232 HCC patients. According to the previous formula, the patients from the GEO and ICGC dataset were divided into high- and low-risk groups. Correlation between the clinicopathologic characteristics and the 7-IRG risk signature in the GEO and ICGC dataset was presented in Additional file 1: Table S2 And S3. Patients in the low-risk group had a better OS than the high-risk group of the GEO and ICGC dataset, consistent with our previous results (Fig. 6a and d). Furthermore, ROC curve analysis based on the 7-IRG risk signature indicated the 1, 3, and 5 years AUC based on the GEO HCC dataset were 0.63, 0.66, and 0.61 (Fig. 6b), and the AUC of 0.64 at 1 year, 0.65 at 3 years, and 0.74 at 5 years based on the ICGC HCC cohort data (Fig. 6e). The 7-IRG also ﻿yielded a better performance than clinical variables in GEO and ICGC dataset (Fig. 6c, 0.711 vs. 0.629, *p* = 0.024; Fig. 6f 0.740 vs. 0.670, *p* = 0.048). The results showed that the 7-IRG risk signature had a robust and efficient performance in predicting HCC patients' prognosis.

### Correlation between the immune-related risk signature and the patients' overall survival

Moreover, we performed a univariable cox analysis to analyze the relationship between OS, clinical variables, and 7-IRG risk signature in the total set (Table 3). This signature had a strong relationship with worse OS in the total set (HR (95% CI) = 2.723 (1.874–3.956), *p* < 0.001, Table 3). In the multivariable cox regression model, after multivariable adjustment by other clinical features (including age, gender, body mass index (BMI), histological grade, clinical stage, TNM stage, AFP, and vascular invasion), the risk group (high vs. low) could also independently predict OS in the total set (HR (95% CI) = 2.002(1.051–3.812), *p* = 0.035, Table 4). The results indicated that the risk group based on the 7-IRG risk signature was a robust and independent prognostic factor.

**Table 3:** Univariate analysis of clinical features and risk score with Cox proportional hazard model

Covariates	Overall survival HR (95%CI)	*p* Value
Age (< 65 vs. ≥ 65)	1.288 (0.904–1.834)	0.161
Gender (female vs. male)	1.183 (0.823–1.7)	0.363
BMI (< 25 vs. ≥ 25)	1.146 (0.792–1.659)	0.470
Histological grade		
G1	1	
G2	1.137 (0.67–1.93)	0.634
G3	1.202 (0.689–2.095)	0.517
G4	1.641 (0.606–4.442)	0.33
Clinical stage		
Stage I/II	1	
Stage III/IV	2.463 (1.691–3.589)	< 0.001
T stage		
T1/2	1	
T3/4	2.594 (1.813–3.710)	< 0.001
AFP (< 300 vs. ≥ 300)	1.018 (0.615–1.685)	0.945
Vascular invasion		
None	1	
Mico	0.833 (0.519–1.337)	0.449
Macro	1.284 (0.516–3.193)	0.591
Risk (high vs. low)	2.723 (1.874–3.956)	< 0.001

**Table 4:** Multivariate analysis of clinical features and risk score with Cox proportional hazard model

Covariates	Overall survival HR (95%CI)	*p* Value
Age (< 65 vs. ≥ 65)	1.418 (0.766–2.625)	0.267
Gender (female vs. male)	0.856 (0.471–1.556)	0.610
BMI (< 25 vs. ≥ 25)	1.495 (0.852–2.623)	0.16
Histological grade		
G1	1	
G2	1.369 (0.391–4.799)	0.624
G3	1.836 (0.528–6.383)	0.339
G4	3.431 (0.695–16.929)	0.130
Clinical stage		
Stage I/II	1	
Stage III/IV	0.995 (0.127–7.817)	0.996
T stage		
T1/2	1	
T3/4	2.176 (0.269–17.610)	0.466
AFP (< 300 vs. ≥ 300)	0.902 (0.469–1.736)	0.758
Vascular invasion		
None	1	
Mico	1.201 (0.616–2.340)	0.590
Macro	3.322 (1.208–9.141)	0.02
Risk (high vs. low)	2.002 (1.051–3.812)	0.035

### Relationship between the immune-related risk signature and clinical features

**Fig. 7:**
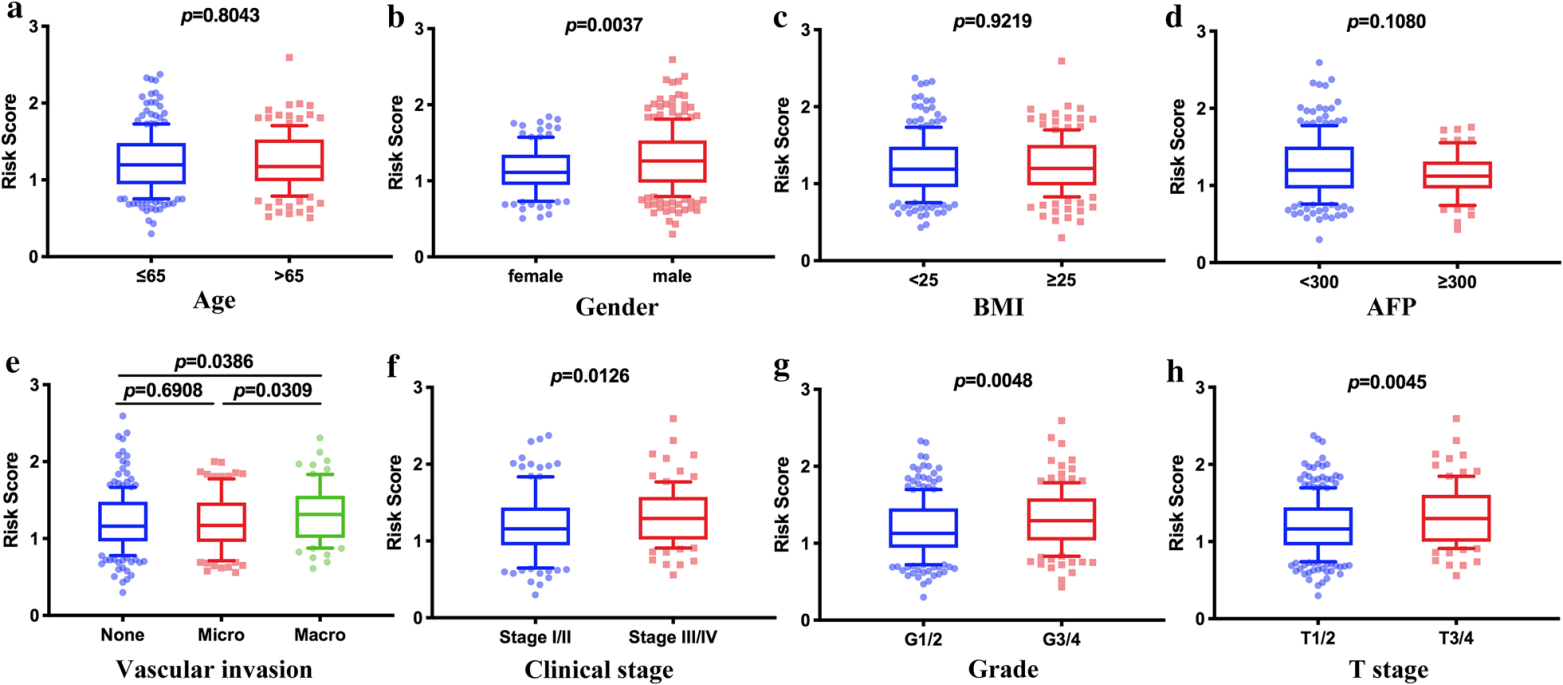
The relationships between the immune-related risk signature and clinicopathological characteristics. **a** Age; **b** Gender; **c** BMI; **d** Serum AFP level; **e** Vascular invasion; **f** Clinical stage; **g** grade; **h** T stage. The *p*-value was indicated in detail

We analyzed the relationships between the risk score and clinical features. The risk score was significantly higher in males, macrovascular invasion, advanced histological grade, clinical stage, and T stage (Fig. 7b, e, f–h). These results demonstrated that the 7-IRG risk score had an essential correlation with clinical subtype classification. Nevertheless, there is no difference between age, BMI, and AFP (Fig. 7a, c, d).

### Alteration of tumor microenvironment associated with the immune-related risk signature

**Fig. 8:**
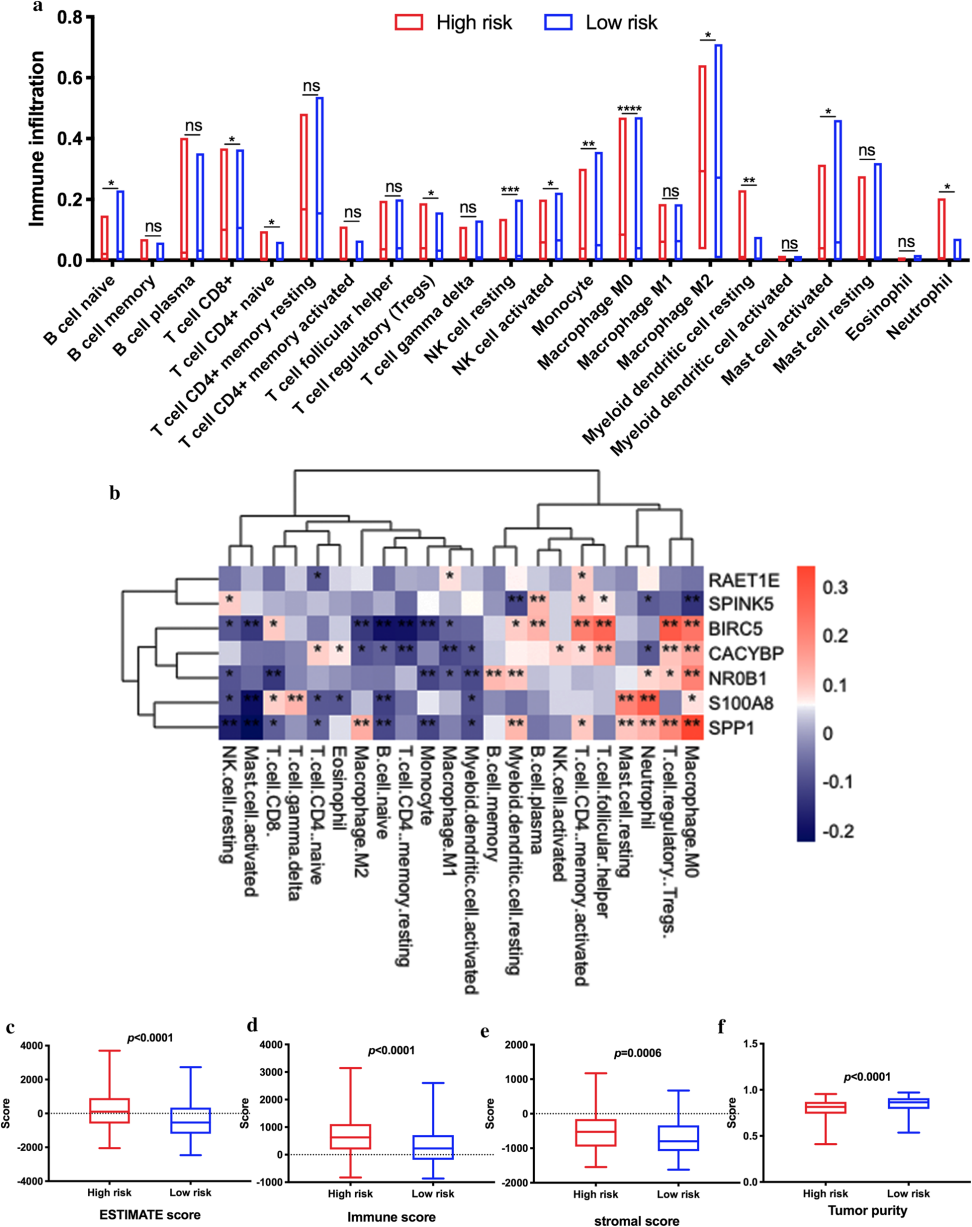
Estimation of TME immune cell infiltration characterization. **a** The relative proportion of 22 tumor-infiltrating immune cells among risk groups. **b** The correlation between 7-IRG signature and each TME infiltration cell type. Red, positive; Blue, negative. The correlation was performed by using Pearson correlation analysis. **c-f** The box plot indicated the difference of estimate score, immune score, stromal score, and tumor purity between risk groups. **c** Estimate score; **d** Immune score. **e** Stromal score; **f** Tumor purity. *p < 0.05; ** *p* < 0.01; *** *p* < 0.001; ns, not significant

To determine the relationship between the 7-IRG risk signature and the tumor microenvironment (TME), We used the CIBERSORT algorithm to estimate the difference of 22 types of tumor-infiltrating immune cells between the low-risk and high-risk group. Additional file 2: Figure S1 presents a heatmap of 22 kinds of immune cell proportions. The abundance of naïve CD4 + T cells, regulatory T cell (Tregs), M0 macrophage, M2 macrophage, resting myeloid dendritic cells, and neutrophil were significantly more enriched in the high-risk group than in the low-risk group. In contrast, naïve B cells, CD8 + T cells, resting NK cells, activated NK cells, monocyte, and activated mast cells were mainly in the low-risk group (Fig. 8a). We performed Spearman correlation analyses to explore the relationships between 7-IRG risk signature and tumor-infiltrating immune cells. The result showed a significant correlation between these IRGs and tumor-infiltrating immune cells (Fig. 8b). Notably, we noted that M0 macrophages, Tregs, and activated CD4 + memory T cells were positively correlated with the 7-IRG signature. Among the 22 types of immune cells, the relative proportion of resting NK cells, activated mast cells, naïve B cell, monocyte, and activated myeloid dendritic cells were negatively correlated with the 7-IRGs.

Additionally, We applied the ESTIMATE algorithm to calculate the estimated score, immune score, stromal score, and tumor purity, representing the tumor environment [30]. We found these scores were significantly increased high-risk group (Fig. 8c–e). On the contrary, tumor purity was decreased in the high-risk group (Fig. 8f).

### The association between immune-related risk signature and immune checkpoints in HCC

**Fig. 9:**
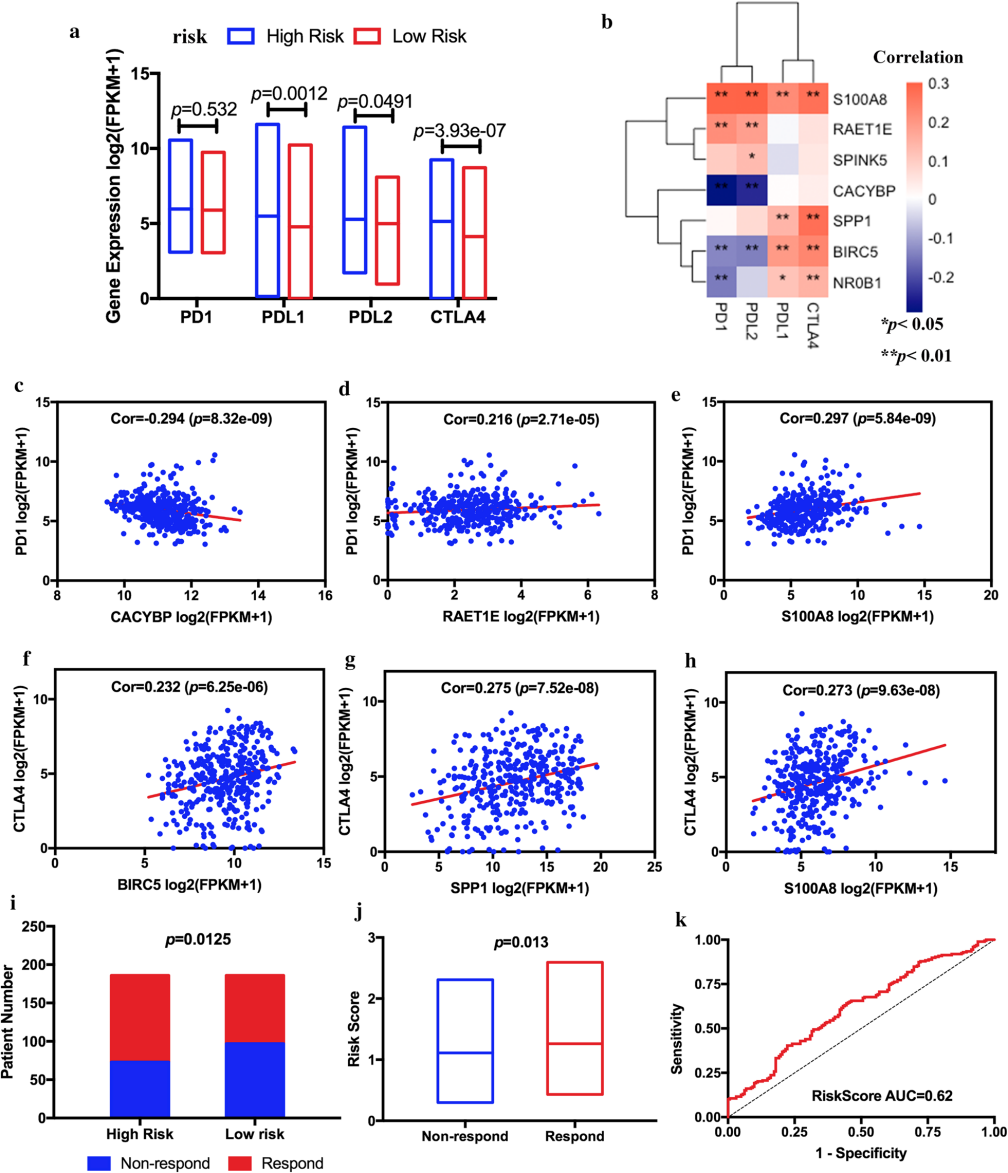
Evaluation of the 7-IRG signature in indicating immunotherapeutic benefit in HCC patients. **a** The different levels of 4 immune checkpoint molecules of HCC tissue among risk groups. **b** The correlations between 7-IRG signature and the 4 immune checkpoint molecules. Red, positive; Blue, negative. The relationships between the immune-related risk signature and PD1 expression. **c** CACYBP; **d** RAET1E; **e** S100A8. **f–h** The relationships between the immune-related risk signature and CTLA4 expression. **f** BIRC5; **g** SPP1; **h** S100A8. The correlation was performed by using Pearson correlation analysis. **i** The distribution of immunotherapeutic response between risk groups in HCC patients. **j** The difference in risk score between non-responders and responders. **k** ROC curves for 7-IRG in predicting the immunotherapy response

To determine the relationship between the 7-IRG risk signature and the immune checkpoint molecules, We confirmed the difference in expression of immune checkpoint molecules in TCGA HCC samples between the low-risk and high-risk groups (Fig. 9a). PDL1, PDL2, and CTLA4 were significantly up-regulated in the high-risk group. Figure 9b showed that the 7-IRG risk signature correlates with the 4 immune checkpoint molecules. The inhibitors of PD1 and CTLA4 are research hotspots in the treatment of advanced HCC. Our results showed that the expression of CACYBP has a significantly negative correlation with the PD1 level (Fig. 9c). RAET1E and S100A8 were positively related to the expression of PD1 (Fig. 9d and e). BIRC5, SPP1, and S100A8 had a positive correlation with CTLA4 (Fig. 9f–h). Furthermore, we utilized the TIDE algorithm to explore whether the 7-IRG could reflect the immunotherapeutic benefit in HCC patients. The detailed output information of TIDE algorithm in TCGA-HCC dataset was shown in Additional files 3. The result showed that the number of immunotherapy responders was significantly higher in high-risk patients (113/186) than low-risk group (89/186) (chi-square tests, *p* = 0.0125) (Fig. 9i). And the risk score was significantly positively correlated with the immunotherapy response (Fig. 9j). The ROC curve depicted that displayed an appropriate predictive effect to ICIs response (Fig. 9k). Above all, these results indicated that ICIs might be more productive for the high-risk group of HCC patients.

### The relationship between immune-related risk signature and mutation profile in HCC

**Fig. 10:**
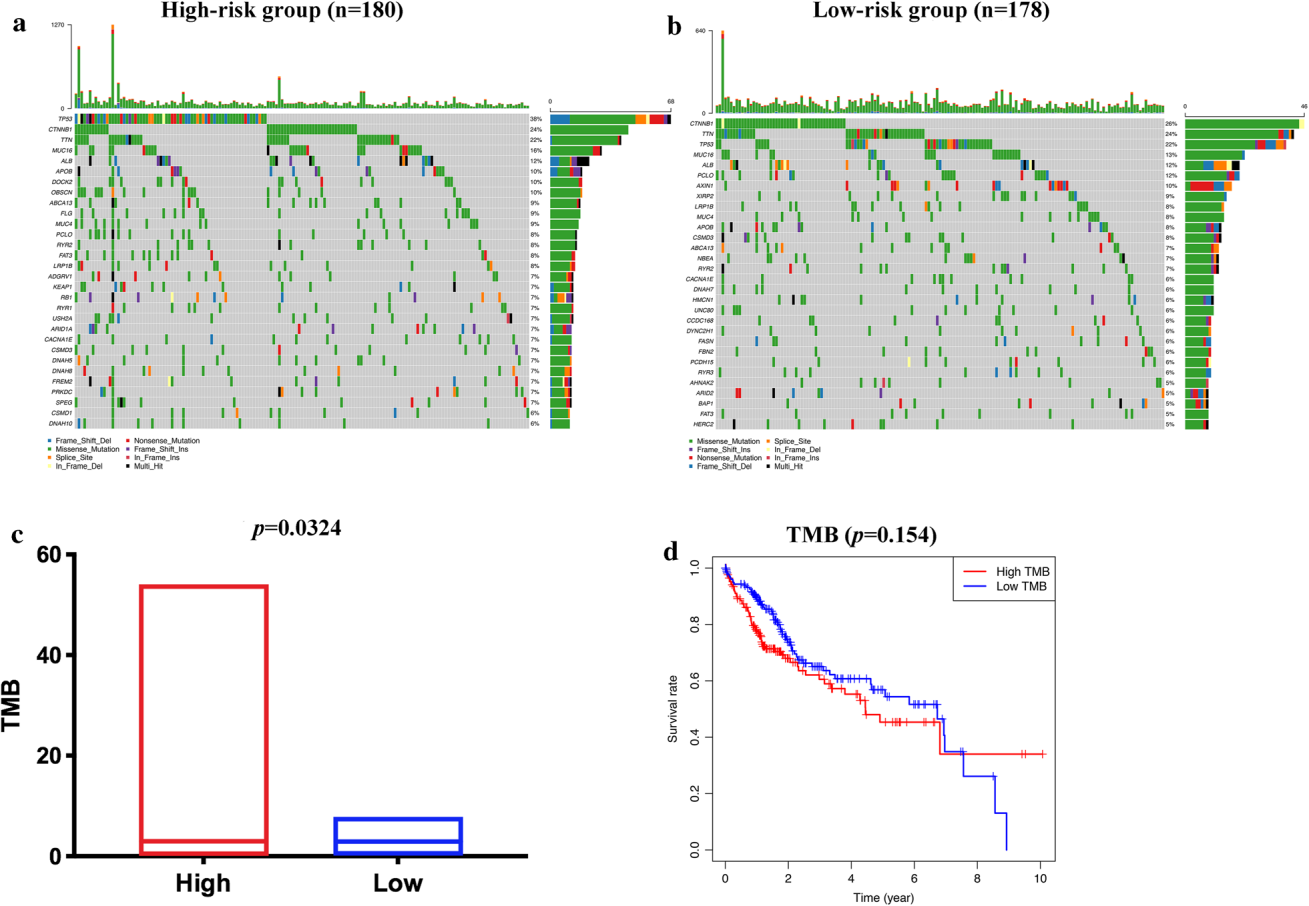
The mutation profile and TMB among low-risk and high-risk groups. **a** Mutation profile in the high-risk group. **b** Mutation profile in the low-risk group. **c** The relationship between the immune-related risk signature and TMB. **d** The association of TMB and overall survival in the TCGA HCC dataset

To assess the relationship between mutation profile and the signature, we further analyzed available somatic mutation data of TCGA HCC patients. Additional file 2: Figure S2 showed a summary of the overall mutation profile of TCGA HCC data. Figure 10a, b depicted the top 20 mutated genes in two risk groups. TP53, CTNNB1, TTN, MUC16, ALB, APOB, ABCA13, MUC4, PCLO, RYR2, FAT3, LRP1B, CACNA1E were the common frequently mutated genes in the low-risk and high-risk groups. TMB was significantly higher in high-risk patient (*p* = 0.0324; Fig. 10c). We also demonstrated that TMB was not associated with OS (*p* = 0.154; Fig. 10d).

## Discussion

Patients with advanced HCC are at substantial risk of death, and chemotherapy remains unsatisfied with a survival benefit. Evidence has proved the potential of immune checkpoint inhibitors (ICIs) in HCC treatment [8, 35–37]. However, only a small portion of HCC patients achieved a therapeutic effect from ICIs. Therefore, it is necessary to explore novel biomarkers for the prognosis and immunotherapeutic responses of HCC. Due to the inhomogeneity of immune response and tumor biology, an onefold biomarker unlikely will provide an accurate prediction of clinical outcomes and response to immunotherapy [38]. Thus, the integrative analysis of the genome and transcriptome data of HCC and immune response parameters might provide us comprehensive view for precise prediction. It draws our attention to dig for the immune-related signature by combining genomic data with computational methods. The immune-related signature may contribute to HCC patients' prognosis and distinguish which patients will be the best immunotherapy candidates.

Therefore, the beginning of our research was to investigate the differentially expressed immune-related genes (DE IRGs) between HCC and normal liver tissues in the TCGA HCC dataset. Then we submitted these DE IRGs to LASSO cox regression analysis to establish a 7 immune-related genes (7-IRG) signature, which stratified HCC patients into the high-risk and low-risk groups with significantly different OS. Six out of seven DE IRGs (S100A8, BIRC5, CACYBP, NR0B1, RAET1E, SPP1) were associated with high risk, and SPINK5 was a protective factor. Six genes (BIRC5, CACYBP, NR0B1, RAET1E, SPINK5, SPP1) were up-regulated in the liver cancer tissues compared to the normal tissues in the TCGA HCC dataset, and S100A8 was downregulated. We verified the genomic difference between adjacent and tumor tissues. We also compared the expression difference of these 7 IRGs in 3 human liver cancer cell lines with 1 human normal liver cell line, consistent with the results we acquired from the TCGA HCC dataset. S100A8 combining with S100A9 form a heterodimer that promotes immune responses and repair mechanisms [39]. Studies have demonstrated that S100A8/A9 facilitates HCC cell survival, proliferation, and invasion in vitro. Their ablation impairs tumor growth due to reduced tumor cell proliferation [40, 41]. CACYBP is up-regulated in HCC compared to normal liver tissues and is related to poor prognosis in HCC patients. It contributes to the development and progression of HCC and may serve as a promising therapeutic and prognostic biomarker [42]. RAET1E belongs to a ligand family for NKG2D in humans and can produce a soluble, 35-kDa protein (named RAET1E2) in tumor cells. Researchers found that incubating NK cells with recombinant RAET1E2 protein decreased the surface expression of NKG2D and reduced the cytotoxicity of NK cells to liver cancer cells, HepG2 cells. In other words, RAET1E might impair NKG2D-mediated NK cell cytotoxicity to tumors [43]. Secreted phosphoprotein 1 (SPP1) is overexpressed during the development and progression of different cancers and might act as a potential prognostic biomarker and therapeutic target [44]. SPP1 is one of the signature genes elevated in HCC tissues and closely related to the tumor process [45]. Despite BIRC5, NR0B1, and SPINK5 have not been previously mentioned for their prognostic value in HCC patients, and these remaining genes could act as potential biomarkers.

The 7-IRG signature presented robust prediction capability in the training, testing, total sets. HCC patients in the high-risk group had shorter OS than those in the low-risk group. Moreover, the 7-IRG signature was externally validated in GEO and ICGC HCC datasets. Our results proved that the7-IRGs risk signature had good reproducibility and robustness in prognosis prediction for HCC patients. Because of a heterogeneous disease with lots of clinicopathological characteristics and risk factors, we should perform a stratification analysis to determine whether the 7-IRG was an independent risk factor. Univariate and multivariate Cox regression analysis demonstrated that the 7-IRG signature remained an independent prognostic factor for OS in HCC patients, which explained that the signature is a firm predictive tool. In terms of the clinical utility, the risk signature has a substantial correlation with vascular invasion, clinical stage, histological grade, and T stage, which demonstrated that the risk score calculated by the signature was significantly higher in advanced HCC cases.

Besides, the tumor microenvironment (TME) plays an essential role in tumor development. Immune cell infiltration of TME in situ was considered a valuable indication for the prognosis and immunotherapy response in cancers according to the clinical trials with ICIs [46]. Recently, multiple computational methods were also invented to assess the abundance of immune cell infiltration based on immune genome data of tumor tissue. We used the CIBERSORT algorithm to estimate the abundance of 22 kinds of immune cells in each HCC sample. The results showed that macrophage M0, macrophage M2, Tregs, naïve CD4 + T cells, and neutrophils were more enriched in the high-risk group. And the data from the ESTIMATE method showed that the 7-IRG signature was positively correlated with the immune scores, stromal scores, and estimate scores and negatively correlated with tumor purity, which could represent the higher infiltration levels of stromal/immune cells in the TME of the high-risk group. Tregs suppress the immune response via various mechanisms. They can suppress both adaptive and innate aspects of the anti-tumor immune response in a TGFβ-dependent manner by inhibiting CD8 + T cells and NK cells, two of the immune system's primary anti-tumor weapons [47, 48]. In HCC, tumor-associated neutrophils combined with CCL2 and CCL17 promote the growth, progression, and resistance to sorafenib. And they also recruit macrophages and Treg cells into TME, thereby contributing to the formation of an immunosuppressive microenvironment [49, 50]. Tumor-associated macrophages (TAMs) subpopulation in HCC is predominantly of the M2 subtype, an essential promoter for tumor initiation and progression [51]. TAMs can produce various chemokines, such as CCL17, CCL18, and CCL22, which attract Treg cells to tumor sites, thereby impeding cytotoxic T cell activation [52, 53]. In our study, the high-risk group was filled with immunosuppressive cells such as Treg, M2 macrophages, producing the immunosuppressive microenvironment to hamper CD8 + T cells' activation NK cells for eradicating the tumor cells. Thus, the infiltration of CD8 + T cells and NK cells in the high-risk group was less than the low-risk group in our study. Besides, we noted that the fraction of resting myeloid dendritic cells (DCs) was higher in the high-risk group and negatively correlated with the 7-IRG risk signature. DCs are the most potent antigen-presenting cells [54] capable of priming T-cells against tumor-associated antigens (TAAs) involved in HCC progression. Based on these characteristics, DC-based Immunotherapy, which stimulates tumor-specific immune responses, has emerged as a promising treatment strategy for HCC [55]. However, some researchers reported high numbers of Plasmacytoid dendritic cells (pDCs) within tumors correlated with high alpha-fetoprotein levels, more significant vascular invasion, advanced tumor-node-metastasis stage, shorter overall survival, and a higher recurrence rate. The increase of intratumoral pDCs was associated with increased infiltration of Foxp3 + regulatory T cells and IL-17-producing cells [56]. In summary, we inferred that the poor prognosis of high-risk HCC patients might be due to this tumor immunosuppressive microenvironment.

Moreover, expression and regulation of immune checkpoint molecules (such as PD-1, PD-L1, PD-L2, and CTLA-4) also act as a crucial role in immune response regulation by suppressing the activation of protective immune cells and promoting immune surveillance [57–59]. Therefore, it is easy to explain why the expression of immune checkpoint molecules was elevated in the high-risk group in our study. Higher expression of immune checkpoint molecules usually benefits more from immune checkpoint inhibitors (ICIs). The 7-IRG risk signature has a good correlation with the expression of immune checkpoint molecules. A study has illustrated that the programmed cell death ligand 1 (PD-L1) was overexpressed on tumor-associated neutrophils from HCC patients. The PD-L1 + neutrophils effectively inhibited T cells' proliferation and activation, but it could be partially reversed by the blockade of PD-L1 [60]. Meanwhile, our results proved that the expression level of PD-L1 is significantly higher in the high-risk group than the low-risk group. Consequently, it expounded the high-risk HCC patients in our research may benefit from PD-L1 inhibitors. Treg cells, which expressed CTLA-4, were also more enriched in the high-risk group and played a vital role in inhibiting anti-tumor immune responses. Treatment with an anti-CTLA-4 antibody might be an effective treatment for high-risk HCC patients. With the help of the TIDE algorithm, we estimate the immunotherapy response in the TCGA-HCC dataset. There are more immunotherapeutic responders in high-risk groups (113/186) than low-risk groups (89/186). And the 7-IRG risk signature was positively correlated with the immunotherapy response. We inferred that the HCC patients in the high-risk group might be more sensitive to ICIs than the low-risk group. These results further indicated that the risk signature was a potent biomarker for predicting the immunotherapy response.

Multiple studies have uncovered that liver cancer cells acquire aggressive characteristics relying on a series of genome changes [61–63]. Tumor mutation burden (TMB) is strictly correlated to the number of neoantigens arising in a tumor and has emerged as a novel potential biomarker in ICI therapy [64–66]. We performed the mutation analysis to investigate the possible mechanisms of the signature's predictive value. Our data showed that the high-risk group has significantly higher TMB than the low-risk group. Consistent with our findings, a study found that higher TMB was positively correlated with the recurrence risk of HCC after radical hepatectomy [67]. The high-risk group has higher TMB, which further proved that ICIs might be more effective in the high-risk group. It was reported that higher TMB was associated with poor prognosis in patients with HCC [67]. Contrary to what was expected and reported, some researchers also demonstrated that high mutation and neoantigen burden do not influence HCC patients' survival who do not undergo Immunotherapy [68]. In our study, the TMB also did not show an impact on the survival of HCC patients.

Nonetheless, some limitations should be noted in this study. Firstly, ﻿because this study is retrospective, our risk signature's capacity for prediction evaluation should be further verified in multicenter clinical trials and prospective studies for better clinical application. Meanwhile, functional and mechanistic investigations on the 7-IRGs in HCC should be additionally performed.

## Conclusion

We established the 7-IRG risk signature based on the expression of immune genes and the immunocyte infiltration levels. The risk signature presented a robust capability to reflect the HCC patient prognosis and might indicate patients' response to immunotherapy. It may provide a deeper understanding and new insights into developing novel immunotherapies for HCC. This model's predictive capability in HCC needs to be further tested for better prognostic stratification and precision treatment.

## Supplementary Information

**Additional file 1: Table S1**. Gene list and immune category for 7-IRG signature**. Table S2.** Correlation of clinicopathologic characteristics and the 7-IRG risk signature in GSE14520 dataset. **Table S3.** Correlation of clinicopathologic characteristics and the 7-IRG risk signature in ICGC-JP-HCC dataset.

**Additional file 2: Figure S1**. Heat map of the 6 immune cell proportions in hepatocellular carcinoma. **Figure S2**. The summary of overall mutation profile of TCGA hepatocellular carcinoma dataset.

**Additional file 3: **TIDE-LIHC.

## Data Availability

Publicly available datasets were analyzed in this study; these can be found in The Cancer Genome Atlas (TCGA) (https://portal.gdc.cancer.gov/), GSE14520 (https://www.ncbi.nlm.nih.gov/geo/), and International Cancer Genome Consortium (ICGC) (https://icgc.org/).

## Abbreviations

HCC: Hepatocellular carcinoma
IRGs: Immune-related genes
TIME: Tumor immune microenvironment
TME: Tumor microenvironment
BMI: Body mass index
DE IRGs: Differentially expressed IRGs
TMB: Tumor mutation burden
PD-1: Programmed cell death protein 1
CTLA-4: Cytotoxic T lymphocyte-associated antigen 4
PD-L1: PD-1 ligand 1
B7-H3: B7 homolog 3
ICIs: Immune checkpoint inhibitors
OS: Overall survival
GO: Gene ontology
KEGG: Kyoto Encyclopedia of Genes and Genomes TAMs: Tumor-associated macrophages
DCs: Dendritic cells
ImmPort: Immunology Database and Analysis Portal
FDR: False discovery rate
LASSO: Least absolute shrinkage and selection operator
AUC: Area under the curve
TNM: Tumor-node-metastasis
MAF: Mutation Annotation Format
ROC: Receiver operating characteristic. TIDE: Tumour Immune Dysfunction and Exclusion
ESTIMATE: Estimation of Stromal and Immune cells in Malignant Tumours using Expression
C-index: Concordance index
