# Supplementary material for: Identification of a prognostic and therapeutic immune signature associated with hepatocellular carcinoma
Source: Cancer Cell Int. 2021 Feb 10;21:98. doi: 10.1186/s12935-021-01792-4 (PMC7877064; doi:10.1186/s12935-021-01792-4)
Supplement: Supplementary file 1 — Additional file 1: Table S1. Gene list and immune category for 7-IRG signature. Table S2. Correlation of clinicopathologic characteristics and the 7-IRG risk signature in GSE14520 dataset. Table S3. Correlation of clinicopathologic characteristics and the 7-IRG risk signature in ICGC-JP-HCC dataset. [file 12935_2021_1792_MOESM1_ESM.doc]

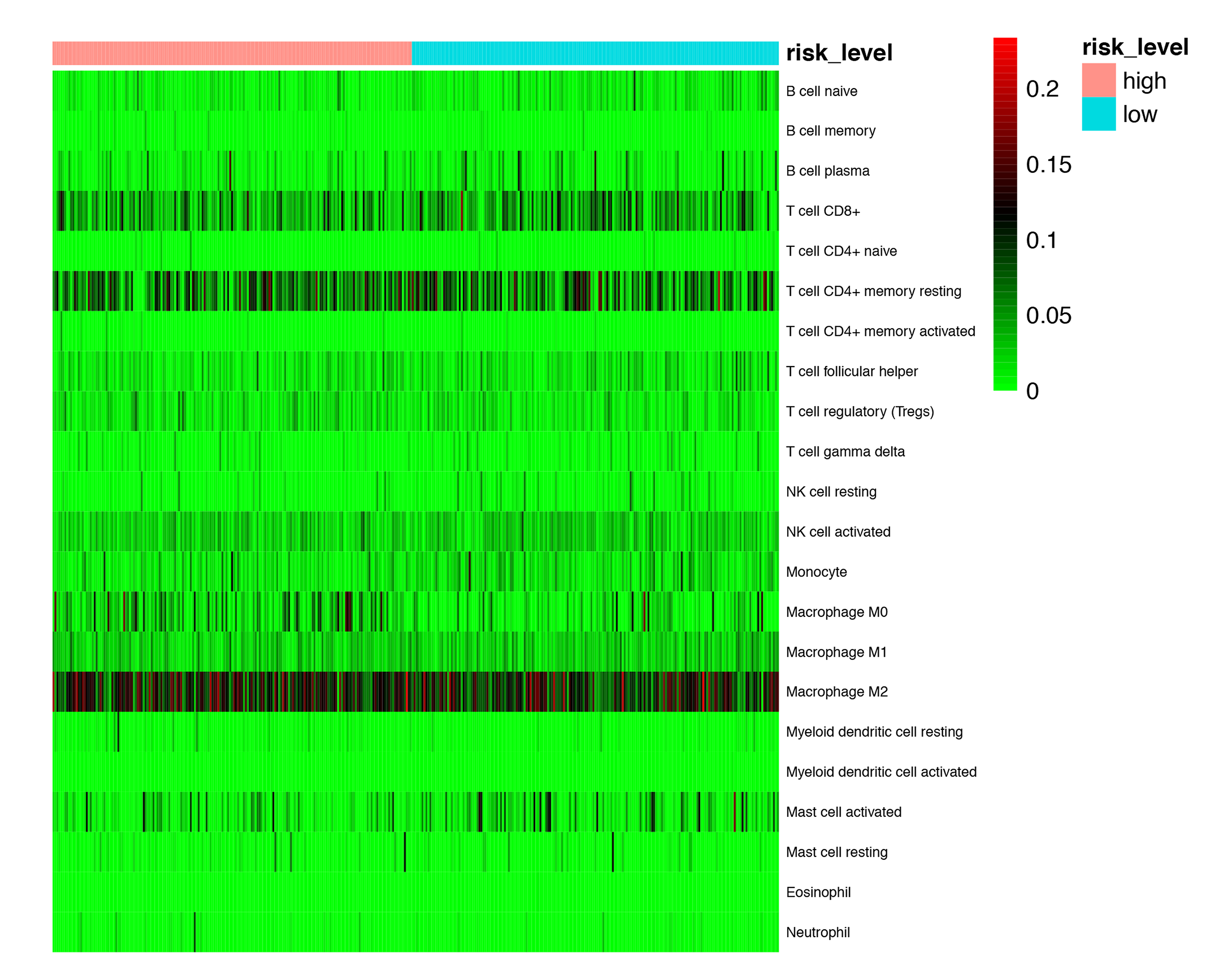


**Figure S1**. Heat map of the 22 immune cell proportions in hepatocellular carcinoma.


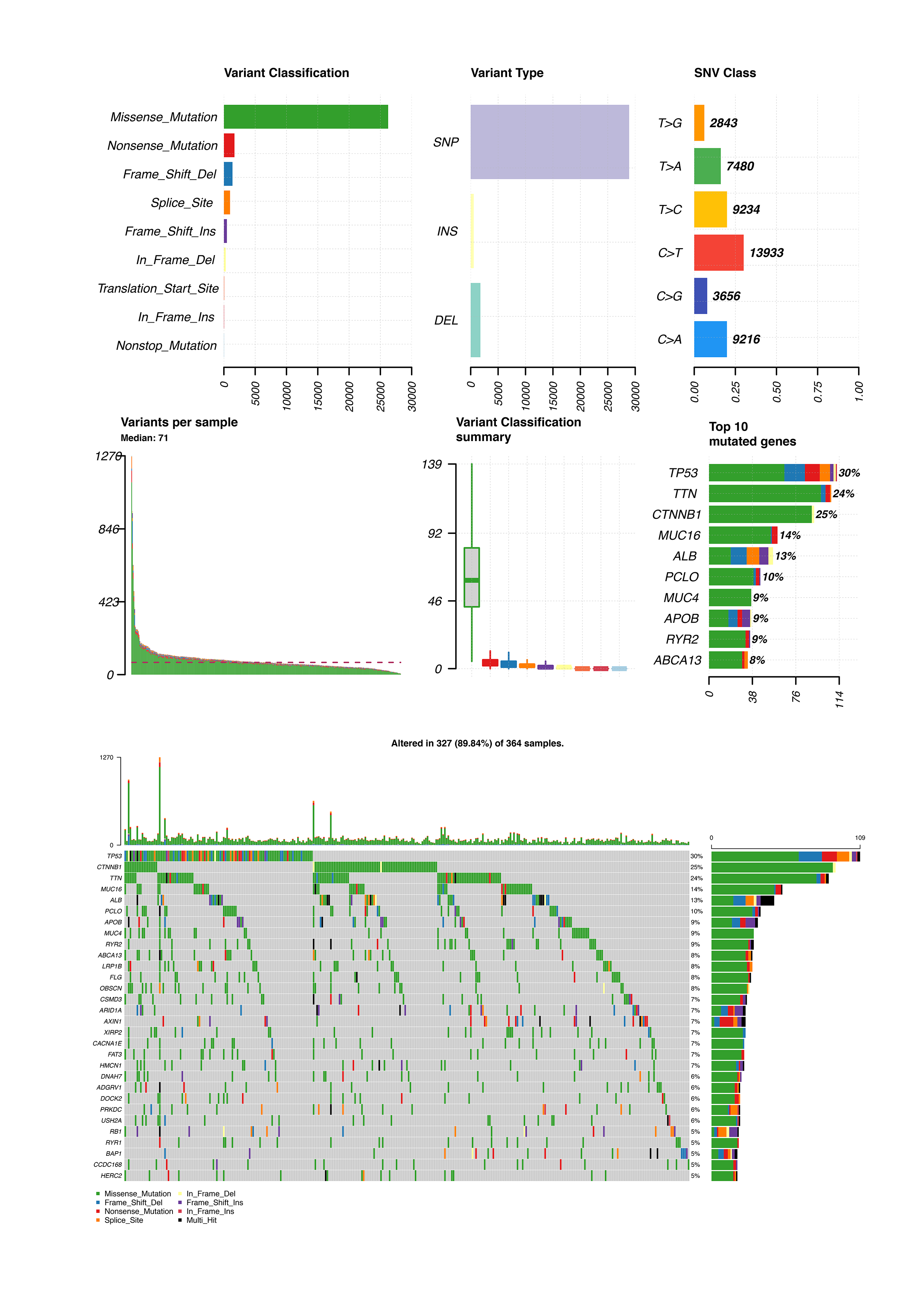


**Figure S2**. The summary of overall mutation profile of TCGA hepatocellular carcinoma dataset.
