# Supplementary material for: Identification of a prognostic and therapeutic immune signature associated with hepatocellular carcinoma
Source: Cancer Cell Int. 2021 Feb 10;21:98. doi: 10.1186/s12935-021-01792-4 (PMC7877064; doi:10.1186/s12935-021-01792-4)
Supplement: Supplementary file 2 — Additional file 2: Figure S1. Heat map of the 6 immune cell proportions in hepatocellular carcinoma. Figure S2. The summary of overall mutation profile of TCGA hepatocellular carcinoma dataset. [file 12935_2021_1792_MOESM2_ESM.doc]

| Gene | ID | Name | Category |
| --- | --- | --- | --- |
| BIRC5 | 332 | baculoviral IAP repeat containing 5 | Antimicrobials |
| CACYBP | 27101 | calcyclin binding protein | Antimicrobials |
| NR0B1 | 190 | nuclear receptor subfamily 0, group B, member 1 | Cytokine Receptors |
| RAET1E | 13250 | retinoic acid early transcript 1E | Antigen Processing and Presentation |
| S100A8 | 6279 | S100 calcium binding protein A8 | Antimicrobials |
| SPINK5 | 11005 | serine peptidase inhibitor, Kazal type 5 | Antimicrobials |
| SPP1 | 6696 | secreted phosphoprotein 1 | Cytokines |

**Table S1**. Gene list and immune category for 7-IRG signature.

**Table S2.** Correlation of clinicopathologic characteristics and the 7-IRG risk signature in GSE14520 dataset.

| **Characteristics** | **High risk(n=17)** | **Low risk(n=225)** | ***p* value** | **method** |
| --- | --- | --- | --- | --- |
| **Risk score** | 1.21 (1.20-1.25) | 0.95(0.82-1.07) | 0.00 | Mann Whitney test |
| **Survival months** | 32.6 (9.75-56.2) | 52.2 (17.7-57.7) | 0.18 | Mann Whitney test |
| **Recurrence months** | 14.3 (4.3-52.25) | 30.9 (10.3-55.2) | 0.10 | Mann Whitney test |
| **Gender** |  |  | 0.38 | χ2 test |
| **Female** | 1 （5.88%） | 30 （13.33%） |  |  |
| **Male** | 16（94.12%） | 195（86.67%） |  |  |
| **Age** |  |  | 0.89 | χ2 test |
| ≤65 | 15（88.24%） | 201（89.33%） |  |  |
| >65 | 2（11.76%） | 24（10.67%） |  |  |
| **ALT(U/L)** |  |  | 0.13 | χ2 test |
| ≤50 | 7（41.18%） | 135（60.00%） |  |  |
| >50 | 10（58.82%） | 90（40.00%） |  |  |
| **Main Tumor Size(cm)** | |  | 0.15 | χ2 test |
| ≤5 | 8（47.06%） | 145（64.73%） |  |  |
| >5 | 9（52.94%） | 79（35.27%） |  |  |
| **Multinodular** |  |  | 0.15 | χ2 test |
| No | 11（64.71%） | 179（79.56%） |  |  |
| Yes | 9（35.29%） | 46（20.44%） |  |  |
| **Cirrhosis** |  |  | 0.21 | χ2 test |
| No | 0 (0.00%) | 19（8.44%） |  |  |
| Yes | 17(100%) | 206（91.56%） |  |  |
| **TNM staging** |  |  | 0.04 | χ2 test |
| I | 5 (29.41%) | 91（43.75%） |  |  |
| II | 4 (23.53%) | 74（35.58%） |  |  |
| III | 8 (47.06%) | 43（20.67%） |  |  |
| **AFP (ng/ml)** |  |  | 0.45 | χ2 test |
| ≤300 | 10 (62.50%) | 118（53.15%） |  |  |
| >300 | 6 (37.50%) | 104（46.85%） |  |  |

**Table S3.** Correlation of clinicopathologic characteristics and the 7-IRG risk signature in ICGC-JP-HCC dataset.

| **Characteristics** | **High Risk(n=179)** | **Low Risk(n=53)** | ***p* value** | **Method** |
| --- | --- | --- | --- | --- |
| **Risk score** | 12.76 (4.52-56.70) | 0.71(0.57-0.90) | 0.00 | Mann Whitney test |
| **Survival days** | 700 (510-1080) | 900 (525-1185) | 0.21 | Mann Whitney test |
| **Gender** |  |  | 0.49 | χ2 test |
| Female | 49 (27.37%) | 12 (22.64%) |  |  |
| Male | 130 (72.63%) | 41 (77.36%) |  |  |
| **Age** |  |  | 0.02 | χ2 test |
| ≤65 | 62 (34.64%) | 28 (52.83%) |  |  |
| >65 | 117 (65.36%) | 25 (47.17%) |  |  |
| **Tumor stage** |  |  | 0.19 | χ2 test |
| stage I | 26 (14.53%) | 10 (18.87%) |  |  |
| stage II | 77 (43.02%) | 29 (54.72%) |  |  |
| stage III | 59 (32.96%) | 12 (22.64%) |  |  |
| stage IV | 17 (9.50%) | 2 (3.77%) |  |  |
| **History of malignancy** |  |  | 0.02 | χ2 test |
| No | 151 (84.36%) | 51 (96.23%) |  |  |
| Yes | 28 (15.64%) | 2 (3.77%) |  |  |
| **Tumor history of first-degree relatives** |  |  | 0.66 | χ2 test |
| No | 112 (66.67%) | 31 (63.27%) |  |  |
| Yes | 28 (33.33%) | 18 (36.73%) |  |  |
